# Allergic diseases of the skin and drug allergies – 2023. Sulfamethoxazole/trimethoprim induced leukocyte count decreased in 79-year-old female patient

**DOI:** 10.1186/1939-4551-6-S1-P110

**Published:** 2013-04-23

**Authors:** Vesna Radovic

**Affiliations:** 1 Pharmacovigilance, STADA Hemofarm A.D., Belgrade, Serbia

## Background

This report was received from a physician via Medicines and Medical Devices Agency of Serbia on 2012-Feb-08.

## Methods

Case report

## Results

A 79-old-year female patient was taking Trimosul (INN: sulfamethoxazole **/** trimethoprim) 400 mg / 80 mg tablets 2 tablets twice daily from 2012-Jan-05 to 2012-Jan-09 for urinary tract infection. On 2012-Jan-09 her total leukocyte count decreased. The suspected drug was withdrawn and her leukocyte count gradually increased. The patient had no previous history of any drug allergy. Laboratory test showed low level of folic acid 1.5 ng/mL (ref. values 2.7-3.4 ng/mL). Concomitant medication was Farin (INN: warfarin) 1 mg daily for pulmonary embolism since 2012-Jan-02 and Ranisan (INN: ranitidine) 150 mg twice daily for gastritis since 2011-Dec-27.

## Conclusions

This case was classified as serious. The causal relationship between sulfamethoxazole **/** trimethoprim and the suspected adverese reaction was assessed as possibly related based on the temporal association and the known safety profile of the drug. The suspected adverse reactions is listed according to the current Reference Safety Information (RSI). This case does not change the overall benefit-risk balance of the medicinal product.

